# Generalized Image Reconstruction in Optical Coherence Tomography Using Redundant and Non-Uniformly-Spaced Samples

**DOI:** 10.3390/s21217057

**Published:** 2021-10-25

**Authors:** Karim Nagib, Biniyam Mezgebo, Namal Fernando, Behzad Kordi, Sherif S. Sherif

**Affiliations:** 1 Department of Electrical and Computer Engineering, University of Manitoba, Winnipeg, MB R3T 5V6, Canada; georgenk@myumanitoba.ca (K.N.); mezgebbk@myumanitoba.ca (B.M.); Behzad.Kordi@umanitoba.ca (B.K.); 2 Manitoba Hydro High Voltage Test Facility, Manitoba Hydro, Winnipeg, MB R3T 1Y6, Canada; snfernando@hydro.mb.ca

**Keywords:** optical coherence tomography, non-uniform discrete fourier transform, redundant samples

## Abstract

In this paper, we use Frame Theory to develop a generalized OCT image reconstruction method using redundant and non-uniformly spaced frequency domain samples that includes using non-redundant and uniformly spaced samples as special cases. We also correct an important theoretical error in the previously reported results related to OCT image reconstruction using the Non-uniform Discrete Fourier Transform (NDFT). Moreover, we describe an efficient method to compute our corrected reconstruction transform, i.e., a *scaled* NDFT, using the Fast Fourier Transform (FFT). Finally, we demonstrate different advantages of our generalized OCT image reconstruction method by achieving (1) theoretically corrected OCT image reconstruction directly from non-uniformly spaced frequency domain samples; (2) a novel OCT image reconstruction method with a higher signal-to-noise ratio (SNR) using redundant frequency domain samples. Our new image reconstruction method is an improvement of OCT technology, so it could benefit all OCT applications.

## 1. Introduction

Optical coherence tomography (OCT) has established itself as an important imaging modality for various medical and industrial applications [1]. Medical applications of OCT include ophthalmology, cardiovascular imaging, and gastrointestinal endoscopy, while its industrial applications include nondestructive testing, material characterization, and microscopic surface profiling. In this paper, we will focus on OCT technology rather than on any specific application.

Because of its higher sensitivity and imaging speed, compared to time-domain OCT, interest in Fourier domain OCT has grown rapidly [2,3,4]. Fourier domain OCT could be further divided into two broad categories: spectral-domain OCT and swept-source OCT (SS-OCT). In spectral-domain OCT, broadband light is backscattered from an object, analyzed by a diffraction grating, and is detected using a line camera [1]. SS-OCT detects backscattered light from an object due to an incident laser whose wavelength is rapidly swept in time [5,6,7,8].

Image reconstruction in Fourier domain OCT typically involves an inverse Fourier transform step applied to its A-scan data. Typical acquisition of this A-scan data, particularly in the case of SS-OCT, leads to non-uniformly spaced samples in the frequency domain. Therefore, it is common to acquire oversampled, i.e., redundant, A-scan data, from which critically sampled, i.e., non-redundant, and uniformly spaced frequency domain samples could be obtained. To avoid the inefficiency of having to acquire redundant A-scan data only to overcome this non-uniform sampling issue, OCT image reconstruction using the Non-uniform Discrete Fourier Transform (NDFT) has been proposed by one of our co-authors [9].

In this paper, we use Frame Theory to develop a generalized OCT image reconstruction method using redundant and non-uniformly spaced frequency domain samples. Our method includes, as special cases, OCT image reconstruction using non-redundant samples, uniformly spaced samples, or both. This method also demonstrates and corrects an important theoretical error in previously published results related to OCT image reconstruction using the Non-uniform Discrete Fourier Transform (NDFT). To demonstrate the advantage of using our corrected OCT image reconstruction method, i.e., a scaled NDFT, compared to using the NDFT, we compare OCT images reconstructed from the same non-redundant and non-uniformly spaced frequency domain samples, using both methods.

Taking an opposite point of view, we also demonstrate for the first time (to our knowledge) the advantages of OCT image reconstruction using redundant, possibly non-uniformly spaced, frequency domain samples to achieve image reconstruction with a higher signal-to-noise ratio (SNR).

This paper is organized as follows: Section 2 gives a brief review of Frame Theory. Section 3 describes our general method for signal reconstruction from redundant and non-uniformly spaced samples and its application to OCT A-scan reconstruction. We also describe an efficient method to compute our corrected reconstruction transform, i.e., a *scaled* NDFT, using the Fast Fourier Transform. Section 4 describes a theoretically corrected OCT image reconstruction from the redundant uniformly spaced frequency domain, and non-uniformly frequency domain spaced samples. Section 5 describes a novel OCT image reconstruction with higher SNR. Section 6 presents our conclusions and future work.

## 2. Brief Review of Frame Theory

Frame Theory is a powerful mathematical tool for the study of redundant linear signal representations. In a linear finite dimension inner product vector space, H, a frame is a generalization of the familiar notion of a basis. Any set of linearly independent vectors ${\left\{\phi_{n}^{basis}\right\}}_{n=1,\;2,\ldots ,\;N}$ in H with dimension, N, could be considered a basis for H.

Let f be an N-dimensional vector fϵ H, 〈·,·〉 be an inner product, and ‖·‖ be its induced norm. Therefore, f could be represented as

(1) $$f=\overset{N}{\underset{n=1}{\sum }}\langle f,\phi_{n}^{basis}\rangle {\tilde{\phi }}_{n}^{basis}$$

where ${\left\{{\tilde{\phi }}_{n}^{basis}\;\right\}}_{n=1,\;2,\ldots ,\;N}$ are the dual basis vectors of ${\left\{\phi_{n}^{basis}\right\}}_{n=1,\;2,\ldots ,\;N}$ that are given by

(2) $$\langle \phi_{n}^{basis},\;{\tilde{\phi }}_{m}^{basis}\rangle =\left\{\begin{array}{c} 1\;\;\;\;n=m\\ 0\;\;\;\;n\neq m \end{array}\right.$$

Therefore, as a special case, the dual basis vectors for any complex-valued orthonormal basis are simply the complex conjugates of its vectors.

In any linear finite dimension inner product vector space, H, with dimension, N, and fϵ H, a vector set ${\left\{\phi_{n}\right\}}_{n\in \Gamma }$ is called a frame if and only if [10]

(3) $$A\|{f\|}^{2}\leq \underset{n\in \Gamma }{\sum }{\left|\langle ,f,\phi_{n},\rangle \right|}^{2}\leq B\|{f\|}^{2}$$

where 0<A≤B are finite values called the frame bounds. We note that the frame condition (3) could be satisfied by ${\left\{\phi_{n}\right\}}_{n\in \Gamma }$ that are either (1) N linearly independent vectors (a basis); or (2) larger than N linearly dependent vectors (a frame). Therefore, a frame in a linear finite dimension inner product space is a generalization of a basis that allows for a potentially redundant linear representation of any vector in this space. In this case, f could be represented as

(4) $$f=\underset{n\in \Gamma }{\sum }\langle f,\phi_{n}\rangle {\tilde{\phi }}_{n}$$

where ${\left\{{\tilde{\;\phi }}_{n}\right\}}_{n\epsilon \Gamma }$ are the dual-frame vectors of ${\left\{\phi_{n}\right\}}_{n\epsilon \Gamma }$ that are given by ${\tilde{\;\phi }}_{n}={\left(\Phi^{*}\Phi \right)}^{-1}\phi_{n}$, where Φ is a matrix whose rows are ${\left\{\phi_{n}\right\}}_{n\in \Gamma }$, and ∗ denotes Hermitian transpose [10].

We note that Equation (4) is mathematically equivalent to the reconstruction of f by orthogonally projecting its frame coefficients vector $\langle f,\phi_{n}\rangle =\;\Phi f$, using the pseudoinverse of Φ that is given by $\Phi^{+}={\left(\Phi^{*}\Phi \right)}^{-1}\Phi^{*}$. Therefore, Equation (4) could also be written as $f=\Phi^{+}\Phi f.$ For *shift-invariant* frames, i.e., when $\phi_{n}$ are shifted versions of a single vector, instead of using ${\tilde{\;\phi }}_{n}={\left(\Phi^{*}\Phi \right)}^{-1}\phi_{n}$ in the time domain, the dual-frame vectors could be obtained easily in the frequency domain. In this case, the Fourier transforms of the dual-frame vectors, $\hat{{\tilde{\phi }}_{n}}\left(k\right)$, where *k* denotes the frequency variable, are given by [10]

(5) $$\hat{{\tilde{\phi }}_{n}}\left(k\right)=\frac{\hat{\phi_{n}}\left(k\right)}{\sum_{n\epsilon \Gamma }{\left|{\hat{\phi }}_{n}\left(k\right)\right|}^{2}}$$

## 3. Signal Reconstruction from Redundant and Non-Uniformly Spaced Samples

### 3.1. Redundant and Non-Uniformly Spaced Samples of Bandlimited Functions as Frame Coefficients

According to the *Nyquist–Shannon* sampling theorem, non-redundant and uniformly spaced samples, obtained at, $t_{n}=nT$, of bandlimited signals with bandwidths, $\left[-\pi /T,\pi /T\right]$, could be viewed as the coefficients representing these signals in an orthonormal basis, ${\left\{\phi_{n}^{basis}\left(t\right)\right\}}_{n\in \mathbb{Z}}$ [10]. The basis vectors are given by

(6) $$\phi_{n}^{basis}\left(t\right)=T^{-1/2}\frac{\sin\left(\pi \left(t-nT\right)/T\right)}{\pi \left(t-nT\right)/T}$$

where *T* is the *Nyquist* sampling interval in the given space of bandlimited functions. Similarly, redundant and non-uniformly spaced samples obtained at $t_{n}$ of bandlimited signals whose bandwidths are included in $\left[-\pi /T,\pi /T\right]$, could be viewed as the coefficients resulting from representing these signals in a frame, ${\left\{\phi_{n}\left(t-t_{n}\right)\right\}}_{n\in \mathbb{Z}}$. The frame vectors are given by

(7) $$\phi_{n}\left(t\right)=\;\Lambda_{n}T^{-1/2}\frac{\sin\left(\pi \left(t-t_{n}\right)/T\right)}{\pi \left(t-t_{n}\right)/T}$$

where $t_{n}$ is the *arbitrary* location of the nth sample, $\Lambda_{n}={\left[\left(t_{n+1}-t_{n-1}\right)/2T\right]}^{1/2}$, and T is once again the *Nyquist* sampling interval in the given space of bandlimited functions. If the maximum sampling distance δ satisfies

(8) $$\delta =\underset{n\in \mathbb{Z}}{\max}\left|t_{n+1}-t_{n}\right|<T$$

then the frame is bounded by $A\geq {\left(1-\delta /T\right)}^{2}$ and $B\leq {\left(1+\delta /T\right)}^{2}$. We note that T/δ represents the oversampling ratio, relative to the *Nyquist* sampling rate, and the scale factor, $\Lambda_{n}$, in Equation (7) accounts for this oversampling and location irregularities of the sampled points.

### 3.2. Frame-Based Reconstruction of an OCT A-Scan

The reconstruction of an OCT A-scan, $u\left(z\right)$, where z is the axial spatial domain variable, from its redundant and non-uniformly spaced frequency domain (*k*-space) samples, is equivalent to reconstructing its Fourier transform, $\hat{u}\left(k\right)$, from its frame coefficients, $\hat{u}\left(k_{n}\right)=\langle \hat{u},\phi_{n}\left(k-k_{n}\right)\rangle$, followed by an inverse Fourier transform. Therefore, using Equation (4)

(9) $$\hat{u}\left(k\right)=\overset{\;}{\underset{n\in \mathbb{Z}}{\sum }}\hat{u}\left(k_{n}\right)\;{\tilde{\phi }}_{n}\left(k-k_{n}\right)$$

For ease of implementation, instead of applying the inverse Fourier transform, we obtain a flipped version of the A-scan by applying the forward transform to Equation (9). By using Equation (5) we obtain

(10) $$u\left(-z\right)=\overset{\;}{\underset{n\in \mathbb{Z}}{\sum }}\hat{u}\left(k_{n}\right)\;\frac{\hat{\phi_{n}}\left(z\right)}{\sum_{n\epsilon \Gamma }{\left|{\hat{\phi }}_{n}\left(z\right)\right|}^{2}}$$

where z is the axial spatial domain variable. The Fourier transforms of the frame vectors corresponding to Equation (7), but in the frequency domain, are given by

(11) $$\hat{\phi_{n}}\left(z\right)=F\left\{\phi_{n}\left(k-k_{n}\right)\right\}=\Lambda_{n}{\hat{\phi }}_{0}\left(z\right)e^{-jk_{n}z}$$

where ${\hat{\phi }}_{0}\left(z\right)$ is the Fourier transform of the unshifted frame vector $\phi_{0}\left(k\right)=\;K^{-1/2}\;\sin\left(\pi k/K\right)/\left(\pi k/K\right)$, where *K* is the *Nyquist* sampling interval in the frequency domain. Substituting Equation (11) into Equation (10), we have

(12) $$u\left(-z\right)=\frac{{\hat{\phi }}_{0}\left(z\right)}{\sum_{n\in \Gamma }{\left|{\hat{\phi }}_{n}\left(z\right)\right|}^{2}}\underset{n\in \mathbb{Z}}{\sum }\Lambda_{n}\left[\hat{u}\left(k_{n}\right)e^{-jk_{n}z}\right]$$

Since the Fourier transform of $\sin\left(\cdot \right)/\left(\cdot \right)$ is a rectangular window, and assuming a finite number of frame expansion coefficients, $N_{f}$, and then replacing the continuous spatial variable, z, in Equation (12) by its corresponding uniformly sampled spatial variable, $z_{s}=0,\;1,\;\ldots ,\;N_{T}-1$, we could approximate $u\left(-z_{s}\right)$ as

(13) $$u\left(-z_{s}\right)≅\frac{1}{N_{T}}\overset{N_{f}-1}{\underset{n=0}{\sum }}\left[\Lambda_{n}\hat{u}\left(k_{n}\right)\right]e^{-jk_{n}z_{s}}$$

where $N_{T}$ is the number of *Nyquist* samples, $N_{T}=\delta N_{f}/T$. Equation(13) is a *scaled* version of the non-uniform discrete Fourier transform (NDFT) at arbitrary points $k_{n}$, rather than the standard NDFT used for OCT reconstruction in [9,11,12]. We note that, compared to the standard NDFT, the extra scale factor $\Lambda_{n}$ in Equation (13) accounts for both oversampling and location irregularities of the sampled points. We will refer to Equation (13) as our theoretically corrected OCT image reconstruction.

### 3.3. Frame-Based Reconstruction of an OCT A-Scan Using the FFT

The reconstruction of an OCT A-scan using the above scaled NDFT, Equation (13), has a computational complexity of $N_{f}f{\left(\log_{2}N_{f}\right)}^{2}$ [9]. However, we could use $O(N_{f}$) operations to enable computing Equation (13) using the Fast Fourier Transform (FFT). We could write Equation (13) as

(14) $$u\left(-z_{s}\right)≅\frac{1}{N_{T}}\overset{N_{f}-1}{\underset{n=0}{\sum }}\left[\Lambda_{n}\;\hat{u}\left(k_{n}\right)e^{-j\;\left[k_{n}-round\left(k_{n}\right)\right]\;z_{s}}\right]e^{-j\;round\left(k_{n}\right)\;z_{s}}$$

where $round\;\left(k_{n}\right)=0,\;1,\;\ldots ,\;N_{T}-1$, is the nearest integer to $k_{n}$ after linearly mapping the measured range of $k_{n}$ to $\left[0,\;N_{f}-1\right]$. We note that Equation (14) is the Discrete Fourier transform (DFT) of the measured A-scan in the Fourier domain, $\hat{u}\left(k_{n}\right)$, multiplied by a complex-valued frequency-dependent scale, $\Lambda_{n}e^{-j\;\left[k_{n}-\;round\left(k_{n}\right)\right]\;z_{s}}$. This resulting DFT could be efficiently computed using the Fast Fourier Transform (FFT) with computational complexity $N_{T}\left(\log_{2}N_{T}\right)$.

## 4. Theoretically Corrected OCT Image Reconstruction from Non-Uniformly Spaced Frequency-Domain Samples

### 4.1. Background and Literature Review

In SS-OCT, a one-dimensional depth profile of an object, i.e., an A-scan, is obtained as a Fourier transform of the acquired data [13]. By combining a series of A-scans, one B-scan image could be obtained, and by combing a series of B-scans, volumetric images could be obtained.

The Fourier inversion step required in SS-OCT is typically implemented using the fast Fourier transform (FFT), a computationally efficient implementation of the discrete Fourier transform (DFT). One major problem of using a DFT-based inversion method is that equally spaced samples in the frequency domain (*k*-space) are necessary, or else the image quality would be compromised [14]. Equally spaced samples in *k*-space cannot be obtained easily because of the nonlinear relationship between the output frequencies of typical swept laser sources and time [15].

Many methods for acquiring uniform samples in *k*-space have been presented in the literature. One method used an auxiliary Mach-Zehnder interferometer (MZI), with a fixed delay between its arms, and a balanced detector. As the wavelength of the laser source is swept, this MZI optical output would be a periodic calibration signal with equidistant maxima and minima in *k*-space [16,17].

A time-domain interpolation scheme was implemented by applying a Fourier transform, followed by a low pass filter, to equidistant samples of the MZI calibration signal. This calibration signal was reconstructed again by applying an inverse Fourier transform on its single-sided spectrum. The resulting complex calibration signal yielded time-dependent wavenumbers, *k*(*t*), that would be fitted by a 3rd order or a higher-order polynomial [18]. Another method that was proposed in [19] used a direct *k*-domain interpolation method based on the spectral phase of the MZI calibration signal. However, MZI based calibration methods add hardware complexity to the system because of the need for an auxiliary interferometer, in addition to the non-efficient use of the optical source since a portion of its power goes to this auxiliary interferometer. A different technique to obtain real-time uniform samples from *k*-domain interpolations used a simple software-based calibration mask [20]. This technique could simultaneously compensate for system dispersion, using generated noise residuals, without elaborate numerical or hardware requirements. A recent method corrected for nonlinear *k*-sampling, in addition to dispersion mismatch in the system, was proposed in [21]. It extracted two calibration vectors to enable numerical resampling for *k*-linearization and phase correction for dispersion compensation.

In [9], one of our co-authors proposed an image reconstruction method for SS-OCT based on the standard NDFT. Compared to interpolation-based image reconstruction methods, this NDFT-based is computationally more efficient, thereby, is more practical [11,12]. However, because this method was not derived earlier from the first principles, it lacks a scale factor that would compensate for the irregularity of samples in the frequency domain. We corrected this important theoretical error in this paper, as shown in Equation (13).

To demonstrate the validity and performance of our *scaled* NDFT based image reconstruction method, in the following sections, we compare its SS-OCT image reconstruction results to results obtained by using the standard NDFT.

### 4.2. Generalized Reconstruction Results Using Synthetic SS-OCT Samples

To quantitatively compare the performance of our *scaled* NDFT based image reconstruction method with the performance of the standard NDFT reconstruction, we applied both methods to non-uniformly spaced, possibly redundant, frequency domain samples that we synthetically generated from two OCT images (512×1000 pixels) of human retinas. These two images are from a public dataset of Fourier-domain OCT images that were obtained from either control subjects or subjects with intermediate age-related macular degeneration [22].

We generated these synthetic samples by Fourier transforming the A-scans of this OCT image and oversampling them by 20 times. Then, non-uniformly spaced, possibly redundant, samples were obtained by non-uniformly selecting samples from these 20 times oversampled Fourier-domain A-scans. The original OCT image of the human retina was then reconstructed from these synthetic samples using both the standard NDFT and our *scaled* NDFT methods.

**Figure 1:**
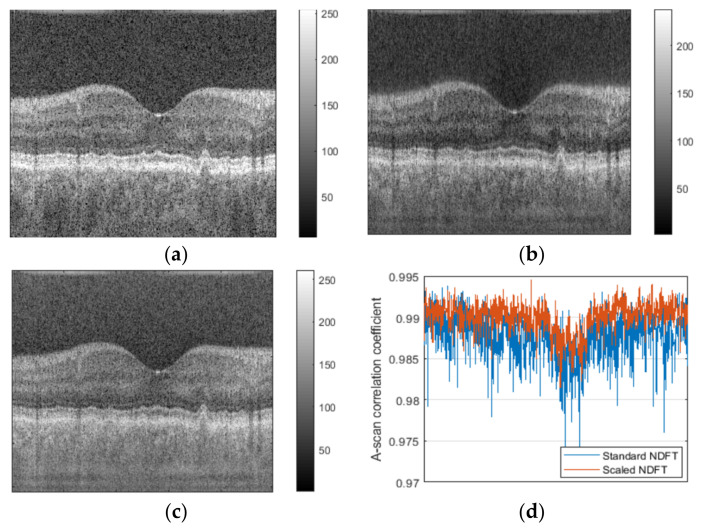
(**a**) Original OCT image of a human retina; (**b**) reconstructed image using standard NDFT (without scaling); (**c**) reconstructed image using our scaled NDFT; (**d**) correlation coefficients between corresponding A-scans of the original image and each reconstructed image.

**Figure 2:**
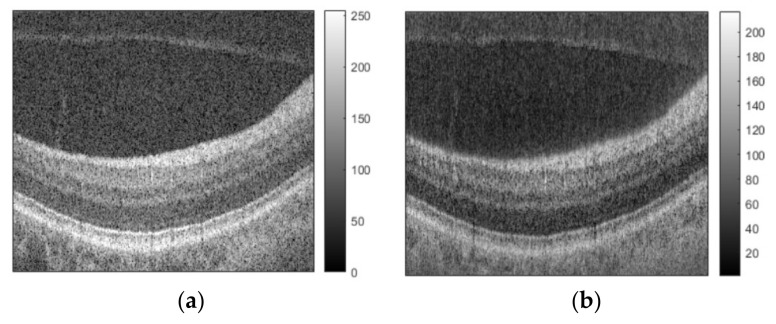

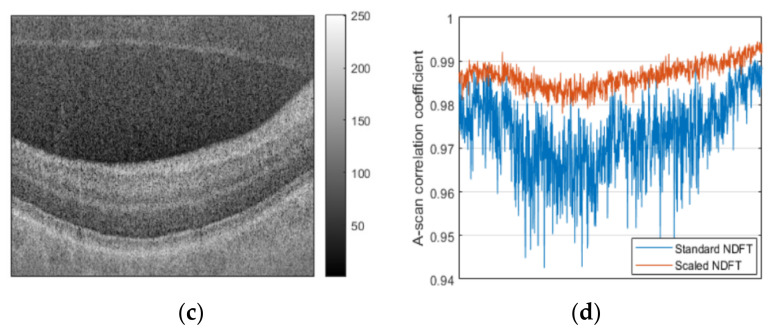
(**a**) Original OCT image of a human retina; (**b**) reconstructed image using standard NDFT (without scaling); (**c**) reconstructed image using our scaled NDFT; (**d**) correlation coefficients between corresponding A-scans of the original image and each reconstructed image.

Figure 1a and Figure 2a show the original OCT images of a human retina. Reconstructed images obtained by applying the standard NDFT are shown in Figure 1b and Figure 2b, while reconstructed images obtained by applying our *scaled* NDFT to the same non-redundant and non-uniformly spaced synthetic OCT samples are shown in Figure 1c and Figure 2c. Figure 1d and Figure 2d show correlation coefficients between corresponding A-scans of the original images and different reconstructed images.

From Figure 1 and Figure 2, we note that, compared to the images reconstructed using the standard NDFT, the images reconstructed using our *scaled* NDFT appear more similar to their original OCT images. This is quantitatively confirmed by the average value of the correlation coefficients between corresponding A-scans in the original images, and the images reconstructed using the standard NDFT (Figure 1 average value = 0.9889 and Figure 2 average value = 0.9733) and using our *scaled* NDFT (Figure 1 average value = 0.9905 average and Figure 2 average value 0.9867). This is a quantitative demonstration of the benefit of using our *scaled* NDFT for OCT image reconstruction.

### 4.3. Generalized Reconstruction Results Using Measured SS-OCT Samples

**Figure 3:**
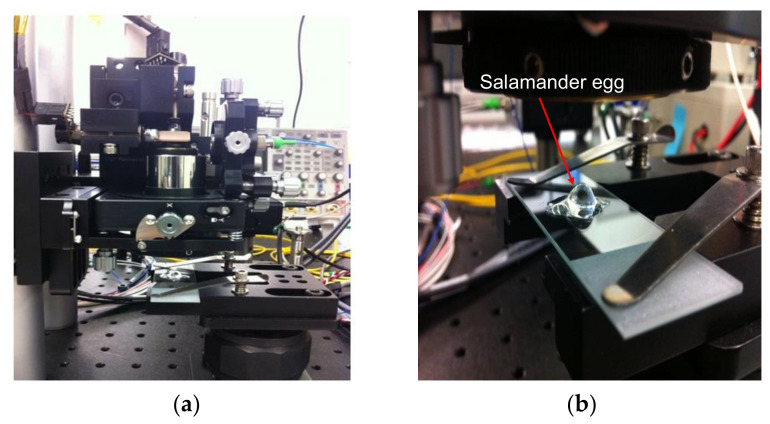
(**a**) Our experimental SS-OCT; (**b**) Axolotl salamander egg at the sample arm.

To qualitatively compare the performance of our scaled NDFT based image reconstruction method with the performance of the standard NDFT reconstruction, we applied both methods to non-uniformly spaced, possibly redundant, frequency domain samples that we experimentally obtained from imaging an Axolotl salamander egg using our SS-OCT system shown in Figure 3. Axolotl salamanders are important lab models for studying numerous biological phenomena ranging from tissue regeneration to cancer. In our SS-OCT system, an interferometer is illuminated with light emitted by our wavelength-swept laser source. A 2 × 2 fiber coupler directs 90% of the incoming light into the sample arm and the remaining 10% into the reference arm. The light reflected back from both reference and sample arms is then redirected into another 2 × 2 fiber coupler, where the interference fringes of the two wavefields are detected by a balanced photodetector. This detected analog OCT signal is then converted to a digital signal using a data acquisition board before being sent to a host computer for digital signal processing.

The implementation of our scaled NDFT, Equation (13), requires knowledge of the relationship between the output frequencies of the swept laser source and time. Assuming the wavelength of the laser source, $\lambda_{s}\left(t\right)$, is a third-degree polynomial in time,

(15) $$\lambda_{s}\left(t\right)=\lambda_{0}+at+bt^{2}+ct^{3}$$

where $\lambda_{0}$ is the initial wavelength. The coefficients a,b,c could be obtained by using nonlinear least squares

(16) $$\;\underset{a,b,c}{\underbrace{\min}}\|I_{MZI}\left(t\right)-y_{MZI}{\left(t\right)\|}^{2}$$

to fit the measured MZI calibration signal, $y_{MZI}\left(t\right)$, to its theoretical model given by

(17) $$I_{MZI}\left(t\right)\propto cos\left(\Delta \phi \left(t\right)\right)=\cos\left(\frac{2\pi d}{\lambda_{s}\left(t\right)}-\frac{2\pi d}{\lambda_{0}}\right)$$

where $\Delta \phi \left(t\right)$ is the phase shift due to path length difference, d, between the MZI arms. After obtaining $\lambda_{s}\left(t\right)$ we could easily obtain, $k_{s}\left(t\right)$, from which we could obtain the sampled k-space frequencies to be used in Equation (13).

We start by obtaining the values of the non-uniform *k*-space frequencies used to acquire our A-scans. The following result is for our wavelength-swept laser source (Thor Labs, SL1325-P16). It has a center wavelength of 1325 nm, a wavelength range from 1250 nm–1375 nm, and an average output power of 15 mW. This laser has a built-in MZI clock, i.e., an interference fringe signal whose zero crossings are equally spaced in k-space. We measured this MZI clock using an oscilloscope (Agilent Technologies, MSO-X 3104A).

After measuring the MZI calibration signal, $y_{MZI}\left(t\right)$, of our swept laser source, and using the Gauss-Newton method to solve Equation (16), we have

(18) $$\lambda_{s}\left(t\right)=\lambda_{0}+0.00225t+1.9812\times 10^{-6}t^{2}+1.999\times 10^{-9}t^{3}$$

where $\lambda_{s}$ and t are in nanometres and nanoseconds, respectively. Using $\lambda_{0}$ = 1250 nm, Equation (18) could be approximated as a linear function

(19) $$\lambda_{s}\left(t\right)=1250+0.00225t$$

which we used to obtain the needed sampled *k*-space frequencies, *k_s_*(*t*).

**Figure 4:**
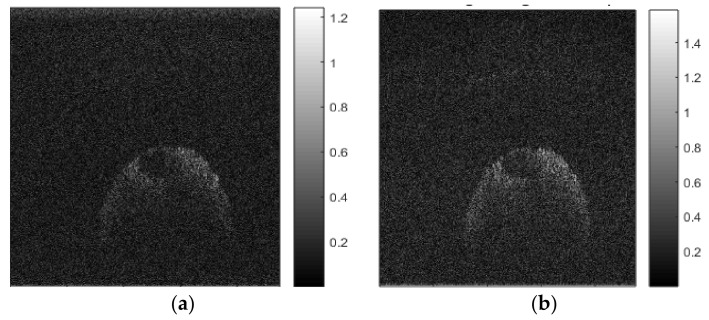
Reconstructed OCT image of a salamander egg using: (**a**) standard NDFT (without scaling); (**b**) our *scaled* NDFT.

Figure 4a shows an image of an Axolotl salamander egg that was reconstructed from non-redundant and non-uniformly spaced SS-OCT samples using the standard NDFT. Figure 4b shows the result of applying our *scaled* NDFT to the same SS-OCT samples.

We note that the image reconstructed using our *scaled* NDFT is clearer overall and has better-defined edges compared to the one reconstructed using the standard NDFT. This demonstrates that our *scaled* NDFT-based OCT image reconstruction is valid and is superior to the current standard NDFT-based reconstruction method.

## 5. Novel OCT Image Reconstruction with Higher SNR Using Redundant Frequency-Domain Samples

### 5.1. Background and Literature Review

In this section, we describe a novel OCT image reconstruction with a higher signal-to-noise ratio (SNR) using redundant frequency domain samples, where we obtain significantly higher peak SNR values for reconstructed images with increased frequency domain sampling rates. Our method could enable faster OCT image acquisition by replacing the sequential acquisition and averaging of a number of similar critically sampled A-scans, a commonly used practice to reduce noise variance in OCT images. Our method is suitable for reducing the variance of any additive zero-mean white noise in OCT data samples.

Noise in OCT systems could be classified as either system noise or speckle noise **(1) System noise:** Typically, due to optical and electrical components of the system, e.g., light source and optical detectors. Shot noise is generated because of random arrival times of photons at the surface of photodetectors [23,24,25,26]. Thermal noise is another important type of system noise that is generated due to the random thermal motion of electrons inside resistive materials [23,27]. We note that system noise is typically modeled as additive noise. **(2) Speckle noise:** When an optically rough object is illuminated with coherent light, e.g., laser, transmitted or reflected light forms a random pattern of dark and bright spots of variable shapes called a speckle pattern [28,29].

Therefore, OCT images are also degraded by speckle noise due to interference of multiple scattered optical fields inside the imaged object. Speckle noise is typically modeled as multiplicative noise; however, one could convert multiplicative noise to additive noise using the Log Stabilizing Transform. This resulting additive noise would not be zero-mean, but the mean of the transformed noisy signal could be subtracted before denoising and added back before inverting the Log Stabilizing Transform [30,31,32].

Much work focused on the study of OCT speckle noise and its reduction. These noise reduction methods use either digital processing or compounding techniques. **(1) Digital processing techniques:** based noise reduction include using an adaptively weighted median filter [33]. By adjusting the filter weights based on the neighborhood statistics of each image pixel, it was possible to suppress noise while preserving important image features. However, its drawbacks include insufficient noise reduction in the case of high noise levels, as well as a loss of image details that could be unacceptable in some applications [34]. Another method to suppress speckle noise is to sequentially apply a set of directional filters to an image and select their maximum output [35]. However, this method could result in a significant loss of image details. An anisotropic diffusion-based noise reduction method, where a family of successively more blurred images is generated through the convolution of the original image with different filters that depend on its local content, was also used for speckle noise reduction [36,37]. Though this method offers better noise suppression and less loss of image detail, it is of limited effectiveness for high speckle noise levels. Total variation regularization was also used for noise reduction [38]. However, this denoising method could lead to a stair-casing effect. Wavelet and other multiscale transformations-based noise reduction methods, where OCT images underwent multiscale decompositions and their coefficients associated with the speckle noise were suppressed, were also used [39,40,41,42]. Nevertheless, such methods could introduce substantial artifacts, dependent on the used wavelet, that could diminish the overall image quality. Other denoising methods using compressed sensing [43] and deep learning [44] have also been reported. **(2) Compounding techniques:** rely on acquiring and averaging different images having uncorrelated or partially correlated speckle patterns [45,46]. Methods to acquire images with uncorrelated speckle patterns include: changing either position or angle of the imaged object or using different optical frequency bands. These methods are referred to as spatial [47], angular [48], and frequency [49] compounding, respectively. However, a drawback of such image compounding is that it could degrade resolution [50]. Other work to maintain the resolution of compounded images, while suppressing noise, has been reported in [51,52]. However, the extra time needed to acquire different images for averaging is always a concern regarding compounding-based noise reduction techniques.

In the following sections, we propose a novel method for SS-OCT noise reduction that requires oversampling, beyond the *Nyquist* rate, of the acquired interferograms followed by our frame-based signal reconstruction described in Section 3. This noise reduction method is conceptually similar to compounding methods, where a number of redundant interferogram samples are averaged to reduce the noise power. However, our oversampling-based noise reduction method has a significant advantage, compared to compounding methods, of saving the extra time needed to sequentially acquire multiple SS-OCT interferograms to construct a single A-scan.

### 5.2. Oversampling-Based SS-OCT Noise Reduction Method

Let the redundant frequency domain (k-space) A-scan samples, $\hat{u}\left(k_{n}\right)$, i.e., the frame coefficients vector, be corrupted by additive zero-mean white noise, $w\left(k_{n}\right)$, with variance $\sigma^{2}$, such that their corresponding noisy coefficients be written as ${\hat{u}}_{noisy}\left(k_{n}\right)=\;\hat{u}\left(k_{n}\right)\;+w\left(k_{n}\right)$. We note that our theoretically corrected OCT image reconstruction, i.e., the scaled NDFT shown in Equation (13), was derived using the dual-frame reconstruction expression given by Equation (4). As mentioned in Section 2, Equation (4) is mathematically equivalent to an orthogonal projection of the frame coefficients vector, Φf, by the pseudoinverse of the frame analysis operator, $\Phi^{+}={\left(\Phi^{*}\Phi \right)}^{-1}\Phi^{*}$. Therefore, the application of our scaled NDFT to k-space A-scan samples is equivalent to

(20) $$\Phi^{+}\left(\hat{u}\left(k_{n}\right)+w\left(k_{n}\right)\right)=\hat{u}\left(-z_{s}\right)+\Phi^{+}\left(w\left(k_{n}\right)\right).$$

Since Equation (20) implements an orthogonal projection, and the noise $w\left(k_{n}\right)$ is assumed white with zero-mean and variance $\sigma^{2}$, the power of the projected noise $\Phi^{+}w\left(k_{n}\right)$ will be bounded by [10]

(21) $$E\left\{{\left|\Phi^{+}w\left(k_{n}\right)\right|}^{2}\right\}\leq \frac{\sigma^{2}}{A}\;\|\phi_{n}{\|}^{2}.$$

Thus, in general, the noise power in the reconstructed A-scan coefficients would be changed from $\sigma^{2}$ to $\sigma^{2}\;\left(\|\phi_{n}{\|}^{2}/A\right)$. For our frame of interest given by Equation (7), the minimum noise variance reduction would occur when $\|\phi_{n}{\|}^{2}=\max\;\left\{\Lambda_{n}^{2}\right\}=\;\delta /T$, and $A={\left(1-\delta /T\right)}^{2}$. Figure 3a shows this theoretical minimum noise reduction (dB) for different values of the oversampling ratio, T/δ. We also show the theoretically expected noise reduction (dBs) due to sequentially acquiring and averaging an integer number of T/δ of the same critically sampled A-scan, where, in this case, the noise variance would be reduced by T/δ.

**Figure 5:**
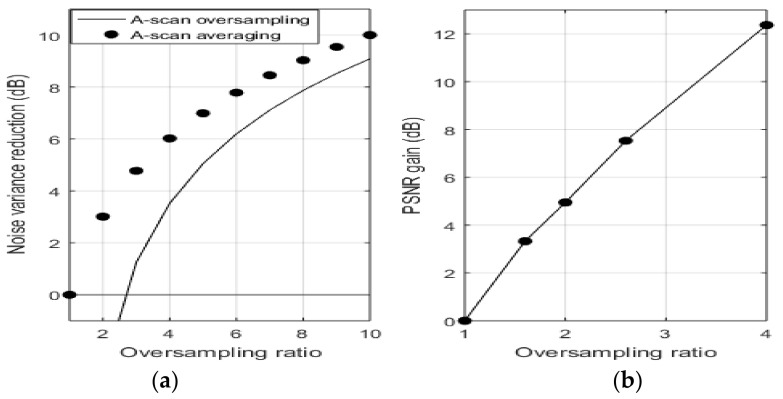
(**a**) Theoretical minimum noise variance reduction for different oversampling ratios; (**b**) PSNR gain in reconstructed salamander embryo images for different oversampling ratios.

From Figure 5a, we note that our oversampling-based SS-OCT noise reduction method, at minimum, would result in significantly reduced noise variance levels. However, these levels would be slightly lower than ones expected by sequentially acquiring and averaging A-scans, and it would be effective only when the oversampling ratio is approximately over 2.3 times. However, our method has the significant advantage of eliminating the relatively long time required to sequentially acquire a number of similar critically sampled A-scans to be averaged. We note that this relatively long sequential acquisition time is equal to the (number of acquired critically sampled A-scans × time to acquire a single A-scan), while our method requires time to acquire a single A-scan only.

### 5.3. Experimental Results

**Figure 6:**
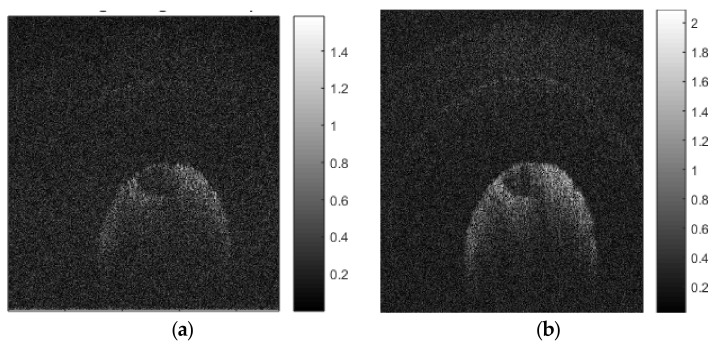
Reconstructed image of a salamander egg using our scaled NDFT with (**a**) 1250 samples; (**b**) 10,000 samples.

Figure 6a,b show images of an Axolotl salamander egg that were reconstructed from 1250 and 10,000 non-uniformly spaced SS-OCT samples, using our *scaled* NDFT algorithm, respectively. Compared to the image reconstructed from the smaller number of samples, the one reconstructed from the larger number of samples appears clearer, with better-defined edges, and the two layers of gel that protect the cell of the embryo could be seen. Therefore, improved image quality, demonstrated by the presence of more visible image details, could be achieved, due to noise reduction, by using a higher sampling rate.

Such improvement of image quality could be quantified by comparing the peak signal-to-noise ratio (PSNR) [53] of different images reconstructed, using our scaled NDFT, from samples with different oversampling ratios. For these PSNR values, shown in Table 1, we used a reference image that was reconstructed, using our scaled NDFT, from 10,000 samples, and we assumed a critical sampling value of 1250 samples, which is a common order of magnitude in typical OCT images.

Figure 5b shows values of PSNR gain (dB) in reconstructed salamander embryo images, computed from Table 1, for different oversampling ratios. On comparing Figure 5b with Figure 5a, we note that our obtained PSNR gain values follow a similar trend as the theoretical minimum noise variance reduction, as expected.

## 6. Conclusions

We used Frame Theory to develop a generalized OCT image reconstruction method using redundant and non-uniformly spaced frequency domain samples that includes using non-redundant and uniformly spaced samples as special cases. We also corrected an important theoretical error in the previously reported results related to OCT image reconstruction using the Non-uniform Discrete Fourier Transform (NDFT). Moreover, we described an efficient method to compute our corrected reconstruction transform, i.e., a scaled NDFT, using the Fast Fourier Transform (FFT). Finally, we demonstrated different advantages of our generalized OCT image reconstruction method by achieving (1) a theoretically corrected OCT image reconstruction directly from non-uniformly spaced frequency domain samples; (2) a novel OCT image reconstruction method with a higher signal-to-noise ratio (SNR) using redundant frequency domain samples. This noise-reduction method could enable faster OCT image acquisition by eliminating the relatively long time required to reduce noise variance by sequentially acquiring and averaging a number of similar critically sampled A-scans. Our new image reconstruction method is an improvement of OCT technology, so it could benefit all OCT applications.

## Figures and Tables

**Table 1:** PSNR of reconstructed salamander embryo images using our scaled NDFT at different oversampling ratios.

Sampling Rate	Critical	1.6×Critical	2×Critical	2.6×Critical	4×Critical
PSNR [dB]	21.33	24.66	26.28	28.86	33.7

## Data Availability

https://people.duke.edu/~sf59/RPEDC_Ophth_2013_dataset.htm.
